# Effect of risperidone on the cravings of patients with methamphetamine use disorder

**DOI:** 10.1192/j.eurpsy.2022.933

**Published:** 2022-09-01

**Authors:** N. Hassan, S. Nazir, U. Sharif

**Affiliations:** 1 Bronx Care Health System, Psychiatry, Bronx, United States of America; 2 Texas Tech University Health Sciences Center, Psychiatry, Lubbock, United States of America; 3 Berkshire Medical Center, Psychiatry, Pittsfield, United States of America

**Keywords:** “Methamphetamine”, “risperidone”, “cravings”, “Psychosis”

## Abstract

**Introduction:**

Methamphetamine associated psychosis has increased globally because of the increased usage of the substance. The use of risperidone is noted to reduce the cravings of methamphetamine in patients who have methamphetamine use disorder. This becomes relevant because the number of patients who are being treated with MAP tends to have high relapse rates. MAP is being treated with different antipsychotics and the treatment protocol is made usually for alleviating the symptoms, a formal treatment regimen for patients with MAP is yet to be developed (Chiang et al 2018; Srisurapanont 2021; Edwards and Mooney 2014)

**Objectives:**

The purpose of this review is to highlight the use of risperidone in reducing the cravings of methamphetamine in patients who have methamphetamine use disorder

**Methods:**

PubMed, SCOPUS and Web of Science literature databases were screened and filtered.With established Inclusion and exclusion criteria, obtained a total of 15578 hits which was refined to 133articles. A total of 10papers were reviewed in detail

**Results:**

Multiple clinical trials have shown that risperidone was effective in lowering drug cravings in methamphetamine use disorder. Along with the effects on craving, risperidone has also been studied for its effect on positive symptoms in patients with MAP (Samei 2016). Risperidone was noted to be effective in reducing positive symptoms.

**Conclusions:**

Risperidone can be effectively used in the acute setting for psychosis and future cravings in the patients. Considering the limited clinical trials and research on risperidone and the cravings of methamphetamine use disorder, studies are needed with longer follow-ups and more samples in the future.

**Disclosure:**

No significant relationships.

